# Features of mental development in preschool children with intellectual disabilities: designing a comprehensive support path

**DOI:** 10.1192/j.eurpsy.2026.11696

**Published:** 2026-07-31

**Authors:** A. Zakrepina, O. Azbukina, Y. Sidneva

**Affiliations:** 1 Psychoeducation, Federal State Budgetary Scientific Institution Institute of Correctional Pedagogics; 2 neurorehabilitation, Clinical and Research Institute of Emergency Pediatric Surgery and Trauma; 3 neuropsychiatry, N.N.Burdenko National Medical Research Institute of Neurosurgery, Moscow, Russian Federation

## Abstract

**Introduction:**

A comprehensive observation of the mental development of preschool children with intellectual disabilities at a medical center revealed numerous variations in mental developmental difficulties. The path to correcting mental disorders was determined by the child’s individual support program and depended on the family’s request for the type of support provided by the medical center’s specialists.

**Objectives:**

to study mental disorders in preschool children with mental retardation in the context of individual training and comprehensive support by specialists of the medical center.

**Methods:**

The sample consisted of 104 children with intellectual disabilities (ages 3 to 5) attending the Medical Center. Of these, 74 children were assigned to the experimental group (EG), which was trained using the center’s integrated solutions algorithm, and 34 children were assigned to the control group (CG), which was trained by an individual specialist at the family’s request.

**Methods:**

parental survey, observation, pedagogical and psychiatric testing, comparative analysis.

**Results:**

Children in the EG showed uniform positive dynamics in mental development in the main areas: 58% of children showed positive dynamics in social-communicative development, cognitive-speech development with persistent mild difficulties in cognitive development; 32% showed positive dynamics in social-communicative and cognitive development with persistent moderate difficulties in cognitive development; 10% showed positive dynamics in social-communicative development and pronounced difficulties in cognitive development and the emotional-volitional sphere.

In the children of the control group, there was an unexpressed dynamics in cognitive development, and only those areas that were identified at the request of parents (speech and communication) were indicative: 38% - with positive dynamics in social and communicative development with persistent difficulties in cognitive development; 35% - with positive dynamics in speech development and persistent mild difficulties in cognitive development and the emotional-volitional sphere; 27% - with unexpressed dynamics in cognitive and social development, and persistent difficulties in the emotional-volitional sphere.

**Conclusions:**

Variability in the mental development dynamics of children with intellectual disabilities in the experimental and control groups determines the most effective approach to correcting mental developmental deviations through comprehensive medical, psychological, and pedagogical support, compared to implementing individual parental requests for support from a specific specialist. Our study aims to further develop and test an action plan for center specialists to address the problem of comprehensive support for children with intellectual disabilities.

**Disclosure of Interest:**

None Declared

